# Evaluation of Pulsed Radiofrequency Denervation in the Treatment of Chronic Facetjoint Pain: An Observational Study

**DOI:** 10.5812/kowsar.22287523.2854

**Published:** 2012-01-01

**Authors:** Gianni Colini-Baldeschi

**Affiliations:** 1 Pain Therapy Unit, Department of Anesthesiology, S. Giovanni-Addolorata Hospital, Rome, Italy

**Keywords:** Low Back Pain, Osteoarthritis, Pulsed Radiofrequency Treatment

## Abstract

**Background:**

Low back disorder is the most common problem in the entire spinal axis. About two-thirds of adults suffer from low back pain (LBP) at some time. Pain generators in the lumbar spine include the annulus of the disc, the posterior longitudinal ligament, a portion of the dural membrane, the facet joints, the spinal nerve roots and ganglia, and the associated paravertebral muscle fascia. There is no doubt that the facet joint is a potential source of chronic LBP. Facet joints are true synovial joints that have a joint space, hyaline cartilage surfaces, a synovial membrane, and a fibrous capsule. Two medial branches of the dorsal rami innervate the facet joints. If conservative measures fail in the treatment of facet joint pain, pulsed radiofrequency (PRF) of the medial branches can be administered.

**Objectives:**

The aim of this observational study was to evaluate the efficacy of PRF in the treatment of lumbar chronic facet joint pain.

**Patients and Methods:**

In this prospective observational study, we selected 300 patients who suffered from lumbar facet joint pain, were referred to the Pain Therapy Department, and underwent PRF treatment of the lumbar medial branches. We analyzed patients with facet joint pain that was unresponsive to conventional treatment, with a positive response to diagnostic medial branch block, who underwent PRF of the lumbar area for 18 months at San Giovanni Hospital of Rome.

**Results:**

Three hundred patients were eligible for the study. After 1 month, 62% of patients (186 patients) reported good pain relief [95% confidence interval (CI) 0.53, 0.7]; 8.6% (26 patients) reported excellent pain relief (95% CI 0.07-0.09); 20. 4% (61 patients) reported poor pain relief (95% CI 0.18-0.22), and 9% (27 patients) reported no pain relief (95% CI 0.08-0.099). The average pain numeric rating scale (NRS) score before the procedure was 6 (range 4-9), decreasing to 2 after the procedure (range 0-4). SF-36 physical and mental parameters improved significantly after the treatment [≥ 1 standard deviation (SD)]. Results after 6 months were similar to those obtained after 1 month.

**Conclusions:**

This study suggests that PRF treatment of the lumbar medial branches provides good pain relief for at least 6 months in 70% of patients who suffer from lumbar facet joint pain.

## 1. Background

Low back disorder is the most common problem in the entire spinal axis. About two-thirds of adults suffer from low back pain (LBP) at some time (1). Pain generators in the lumbar spine include the annulus of the disc, the posterior longitudinal ligament, a portion of the dural membrane, the facet joints, the spinal nerve roots and ganglia, and the associated paravertebral muscle fascia. The lifetime prevalence of chronic osteoarticular pain has been reported to be as high as 60% (2).

Osteoarthritis is a chronic, degenerative joint disease that primarily affects middle-aged and older adults (3). Osteoarthritis is characterized by the breakdown of cartilage in the joint and adjacent bone. As the cartilage wears down, the bone ends may thicken, forming bony growths or spurs that interfere with joint movement. Bone fragments and fluid cysts may be present in the joint space, worsening joint movements (4).

If conservative measures fail in the treatment of facet joint pain, pulsed radiofrequency (PRF) of the medial branches can be used (5). The mechanism of action of PRF is still the subject of debate in the literature. PRF has an effect on pain pathways, reducing nociceptive inputs. The notion that the electrical fields that are generated by PRF can affect neuronal membranes is supported by neurophysiological studies that have demonstrated that PRF alters synaptic signal transmission and causes electroporation (6). Cosman explains that radiofrequency (RF) causes an increase in temperature of the targeted tissue above 45-50°C, and exposure for 20 seconds or more at these high temperatures is lethal to cells.

PRF and continuous radio frequency (CRF) originate from the same underlying physical laws but differ in space, time, and strength of the resultant fields. PRF is characterized as having typically stronger E-fields than CRF and temperature spikes above the average thermal background that can reach 45-50°C. In a frequently adopted practice of holding the average T background at or somewhat below about 42°C, PRF also differs from CRF, in that the spatial extent of continuously elevated temperatures is much less than with CRF (7). Van Zundert et al. (8) demonstrated that PRF on rat dorsal root ganglia at 42 °C for 8 minutes increases c-Fos expression in the dorsal horn. PRF has a selective effect on small unmyelinated fibres (C-fibers), leaving myelinated fibers (A-Delta fibers) unaffected.

PRF is supposed to be less destructive and more reversible than CRF (9). Further research and clinical trials are needed to confirm whether PRF has a nondestructive effect. The heat that is generated by electrical current is dissipated between pulses. In fact, PRF uses radiofrequency current in short (20 ms), high-voltage bursts; the “silent” phase (480 ms) of PRF allows time for heat to subside, generally keeping the target tissue below 42°C.

Cosman, Sluijter, and Rittman (10, 11) formulated the hypothesis that PRF was capable of delivering sufficient RF energy to modulate the electrical field that was insufficient to cause tissue thermocoagulation. Cunen et al. (12-14) showed that although the mean tip temperature remains below neurodestructive levels, PRF has an ablative effect as well, but it is weaker than the effect of a CRF heat lesion. This ablative effect is supposed to be caused by the heat spikes or electric field. The most likely causes of RF-induced neural destruction and injury are heat, high electric field, and high current field.

Heat is the rapid thermodynamic spread of energy of all tissue excitations down to the molecular level, characterized by a global parameter T. Tissue disruption by high E-fields would be more specific than by heat. The E-field induces charges in tissue and produces forces on charged molecular structures, causing them to distort and dislocate. E-field gradients produce dielectrophoretic forces on charged objects, causing stress, distortion, and movement.

Complete reduction can be reached if the nociceptive input is generated in a small, contained area, which occurs when pain radiates from facet joints.

## 2. Objectives

The aim of this observational study was to evaluate the efficacy of PRF in the treatment of lumbar chronic facet joint pain.

## 3. Patients and Methods

Patients who underwent PRF of the lumbar area over 18 months at San Giovanni Hospital of Rome were analyzed. Patients with chronic LBP with sheer nociceptive characteristics and symptomatology that was related to facet joint syndrome and unresponsive to conventional treatment, such as medications and physical therapy, were included in the study. Patients with contraindications to the treatments, such as bleeding disorders, infectious diseases, and neurological impairments, were excluded. Patients were informed about the PRF, which provides relief of pain in many patients with chronic zygapophyseal joint arthropathy.

All patients who entered the study had been extensively investigated [medical history, physical examination, X-ray, magnetic resonance imaging (MRI), x-ray computed tomography (CT)] by the referring physician. They presented with a history of a minimum of 6 months of pain in the lumbar area. Three hundred patients met the inclusion criteria; all of them had been assessed by numeric rating scale (NRS) and SF-36 and reported a positive response to diagnostic block of medial branches (at least 50% relief of pain following diagnostic blockade with 0.5 mL of 2% lidocaine for each medial branch).

In each patient, we treated 3 medial branches that were related to the painful area. Treatment was performed on an outpatient basis. All procedures were performed by fluoroscopy, with the patient in the prone position, using AP, lateral, and oblique views. A 22-gauge needle, 100-mm RF electrode, and active 5-mm tip were used. The cannula tip was placed using the “tunnel vision,” per van Kleef et al. (15). Once the tip needle was at the target, sensory stimulation was carried out at 50 Hz up to 0.5 V to confirm the proximity to the medial branch. Motor stimulation was performed at 2 Hz up to 2 V. The PRF parameters for each medial branch were: 42 °C, 20 ms, 2 Hz, 240, and impedance < 400 Ohm using the Baylis pain management generator. Sedation was not given. Pain was measured with the NRS. The quality of life was measured by SF-36.The therapeutic effectiveness was defined by patients as excellent (pain relief > 80%), good (pain relief > 50%), poor (pain relief < 50%), or ineffective (no pain relief) at 1, 3, and 6 months following the procedure.

## 4. Results

**Figure 1.**
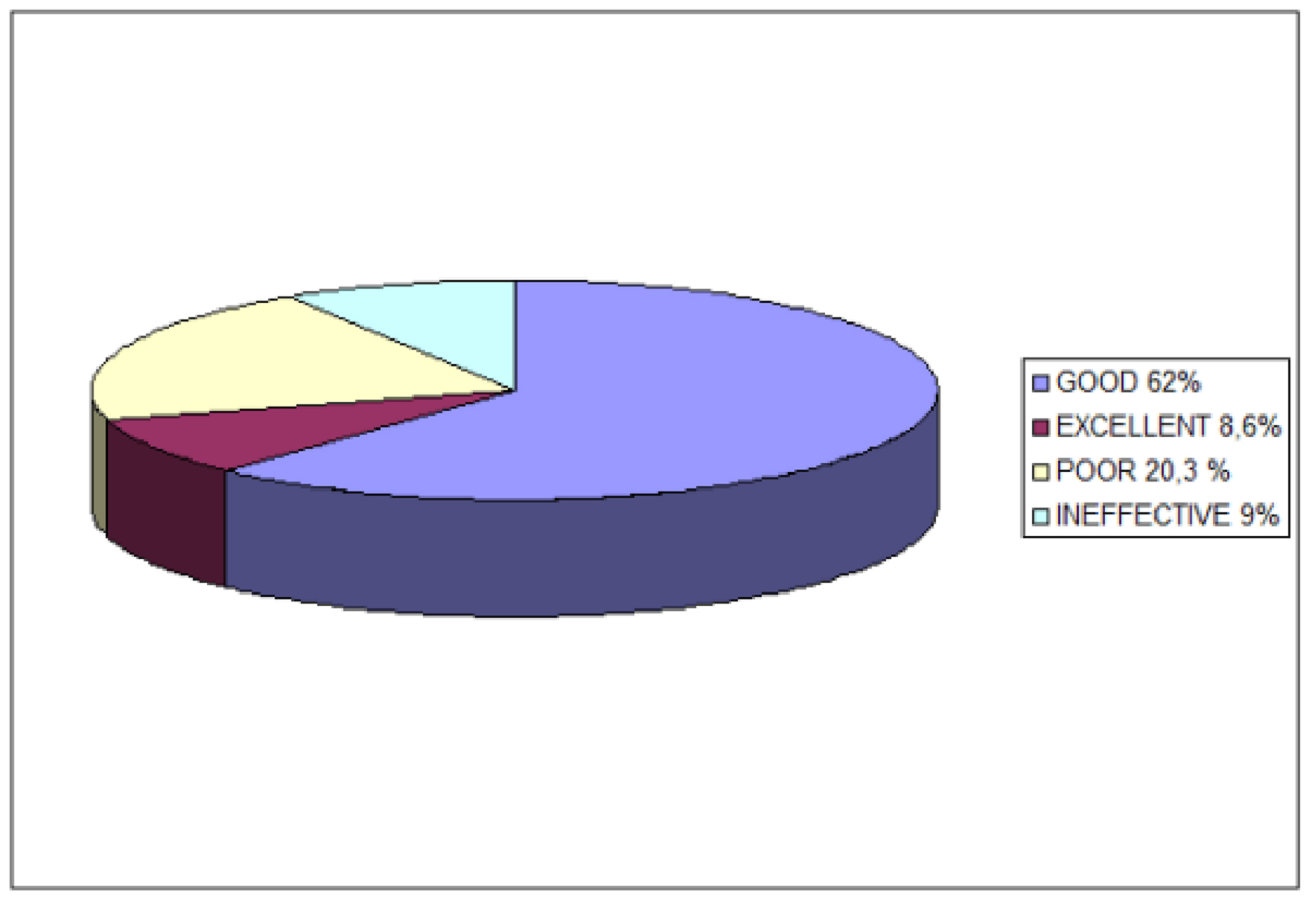
Pain Relief

**Figure 2.**
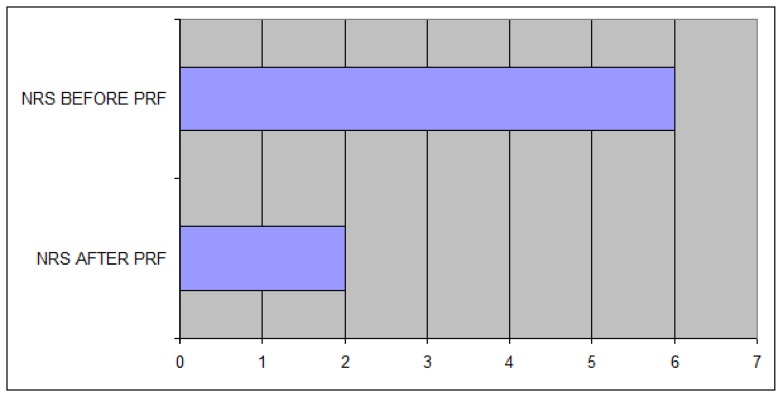
Mean Numeric Rating Scale Before and After Treatment

**Figure 3.**
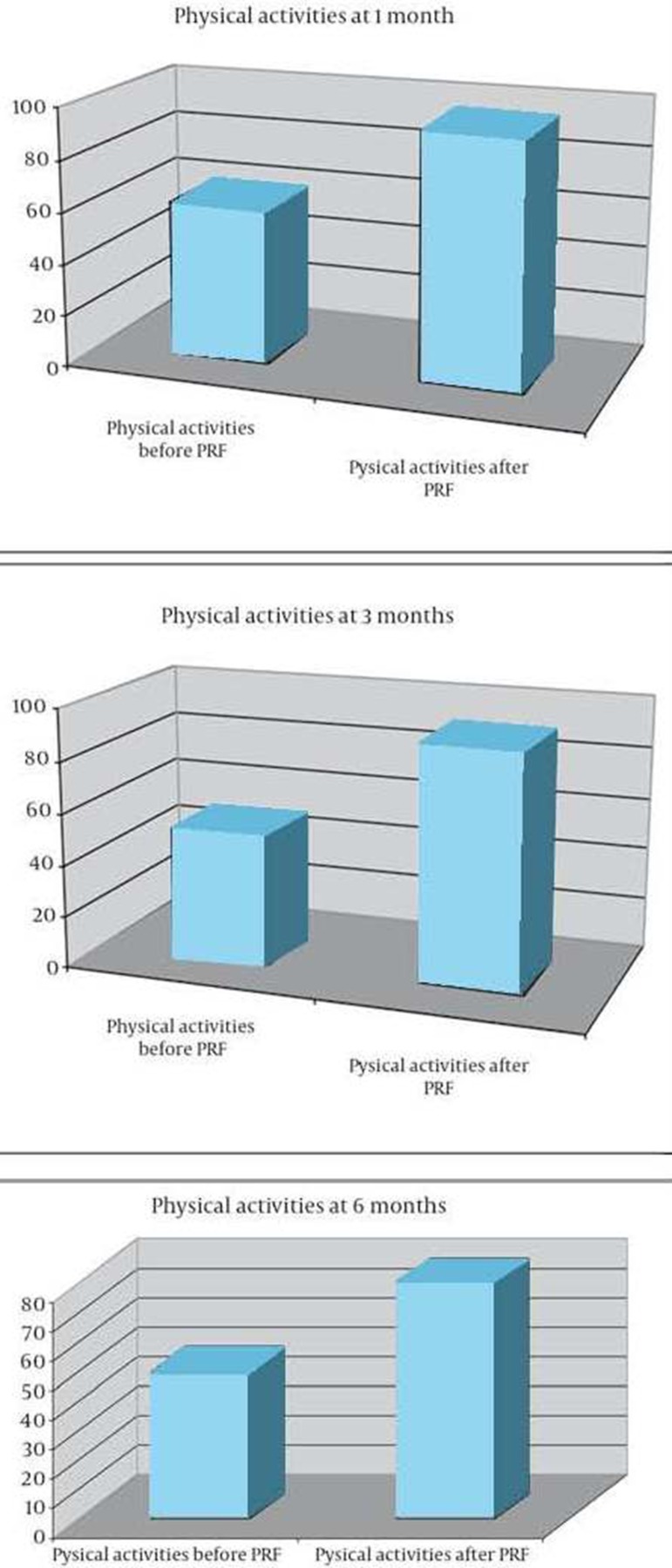
Mean SF-36 Before and After the Procedure-Physical Activities at 1, 3 and 6 Months

**Figure 4.**
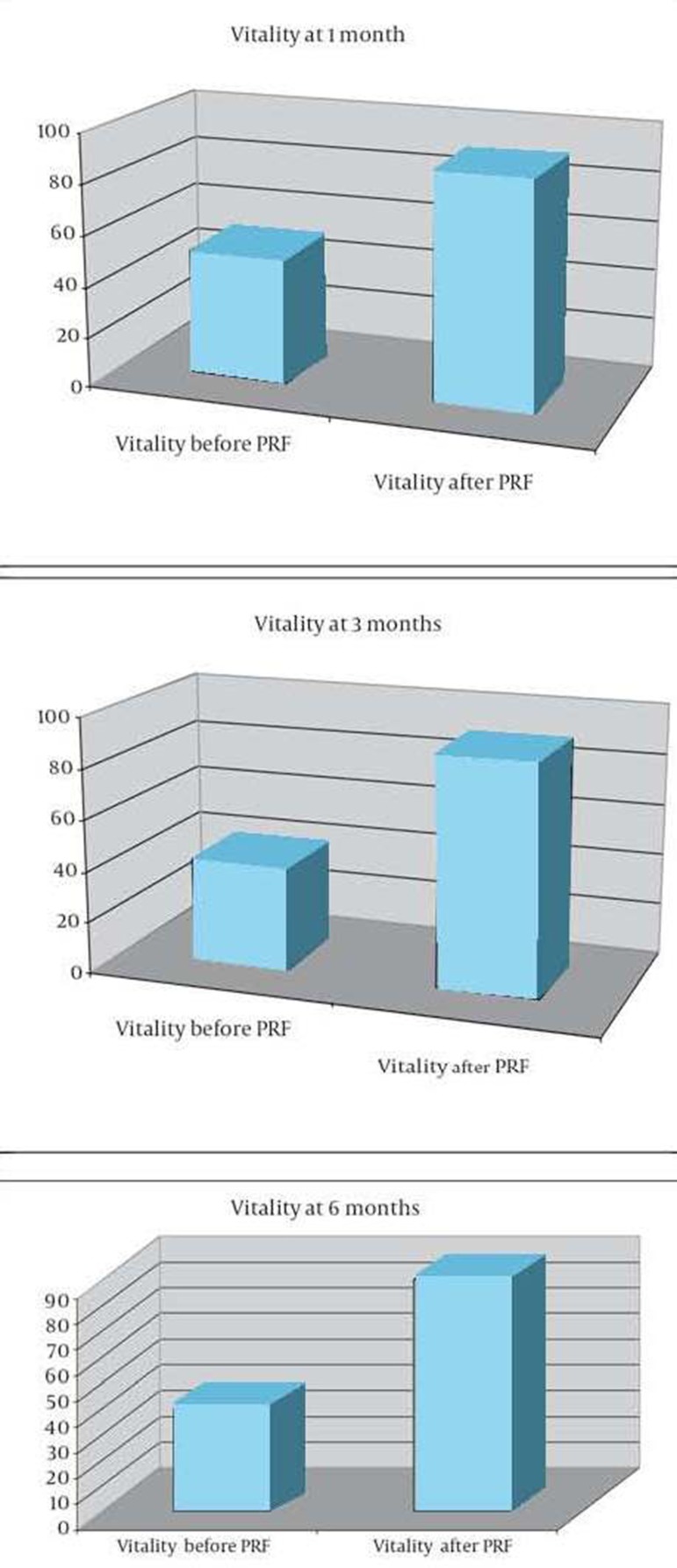
Vitality at 1, 3 and 6 Months

Three hundred patients entered the study. Median age was 68 (range 45-86), and there were 142 males and 158 females. Pain relief was defined by patients at 1 and 6 months after the procedure. Sixty-two percent of patients (186) reported good pain relief [95% confidence interval (CI) 0.53, 0.7]; 8.6% (26 patients) reported excellent pain relief (95% CI 0.07-0.09); 20.4% (61 patients) reported poor pain relief (95% CI 0.18-0.22); and 9% (27 patients) reported no pain relief (95% CI 0.08-0.099) (Figure 1). Median NRS prior to the procedure was 6 (range 4-9); median NRS after the procedure was 2 (range 0-4) (Figure 2). Both physical and mental parameters of the SF-36 improved significantly after the treatment (≥ 1 SD) at 1, 3, and 6 months (Figure 3 and 4). There were no significant significant differences between the results at 1, 3, and 6 months, despite patients reporting more pain relief at 1 and 3 months. Side effects were not noted. During the observational period, patients were not subjected to other pain treatments and did not take any medications.

## 5. Discussion

Various studies have analyzed the efficacy of CRF treatment of the medial branch (16-20). Clinical data on the efficacy of PRF are limited, whereas there is a stronger evidence for CRF, not only with respect to pain relief but also in terms of functional restoration (21-25). Additionally, most reports are retrospective in nature and have involved only small patient groups.

A retrospective study by Mikeladze et al. (26) of 114 patients with cervical or lumbar facet joint pain who were responsive to diagnostic medial branch blocks and subsequently treated with PRF at 42°C for 120 seconds found that 68 patients had significant pain relief (> 50% pain reduction) that lasted an average of nearly 4 months. Lindner et al. (27) carried out a retrospective analysis of 48 patients who were suffering from LBP. All patients were treated with PRF at 42°C for 120 seconds at 2 levels using a 22 electrode with a 5-mm active tip, after successful diagnostic medial branch block. The authors noted a good outcome (> 60% improvement) at the 4-month follow-up.

Tekin et al. (28) performed a randomized, double-blind, study that compared the efficacy of PRF in the treatment of lumbar facet joint pain in 60 patients and found that both CRF and PRF were effective and safe and that pain relief was better than local anesthetic block alone, whereas the duration of pain relief with PRF was less than with CRF.

The use of PRF for the management of patients with zygoapophyseal joint pain was documented in 2 studies (11, 29). A total of 166 patients were treated, with a satisfactory clinical response of 3-6 months. Five retrospective trials reported on patients with different pain syndromes; in total, 343 patients were treated, with satisfactory results (30-33). Fifteen reviews, editorials, letters, and comments that have discussed the use of PRF have been located (34-42). Generally speaking, most authors consider the use of PRF as a minimally neurodestructive alternative option to RF heat lesions due to its potentially better risk/benefit ratio.

Our study was observational, enrolling 300 patients, which is adequte to analyze the efficacy and safety of a technique, such as PRF; however, the limitation of our study is that it was not a randomized controlled trial. There are ethical problems regarding the use of placebo in a controlled trial for patients who suffer from moderate to severe pain; moreover, more findings are needed to carry out a randomized controlled trial, as Gallagher (43) and others (44) claim. Such studies are also troublesome due to the etiological heterogeneity of pain disorders (45).

PRF is not a substitute for CRF under conditions for which thermal RF has an acknowledged and proven efficacy, but PRF is an attractive procedure on several accounts; in fact, PRF is supposed not to be neurolesive. This study suggests that PRF of the lumbar medial branch provides good pain relief 1 month after PRF in 70% of patients, whereas there was a recurrence of pain in 30% of patients. The results after 6 months were similar to those obtained after 1 month. In our experience, PRF is an effective and relatively safe technique, useful for treating facet joint pain that is refractory to conservative treatment. If the pain returns, PRF may be repeated safely. Further studies are needed to evaluate the parameters of PRF, especially temperature. Randomized controlled studies are recommended to compare CRF and PRF.

## References

[1] Deyo RA, Weinstein JN (2001). Low back pain.. N Engl J Med..

[2] Johannes CB, Le TK, Zhou X, Johnston JA, Dworkin RH (2010). The prevalence of chronic pain in United States adults: results of an Internet-based survey.. J Pain..

[3] Loeser RF (2010). Age-related changes in the musculoskeletal system and the development of osteoarthritis.. Clin Geriatr Med..

[4] Buckwalter JA Articular cartilage injuries.. Clin Orthop Relat Res..

[5] Misaggi B, Gallazzi M, Colombo M, Ferraro M (2009). Articular facets syndrome: diagnostic grading and treatment options.. Eur Spine J..

[6] Byrd D, Mackey S (2008). Pulsed radiofrequency for chronic pain.. Curr Pain Headache Rep..

[7] Cosman ER, Cosman ER (2005). Electric and thermal field effects in tissue around radiofrequency electrodes.. Pain Med..

[8] Van Zundert J, de Louw AJ, Joosten EA, Kessels AG, Honig W, Dederen PJ (2005). Pulsed and continuous radiofrequency current adjacent to the cervical dorsal root ganglion of the rat induces late cellular activity in the dorsal horn.. Anesthesiology..

[9] Snidvongs S, Mehta V (2010). Pulsed radio frequency: a non-neurodestructive therapy in pain management.. Curr Opin Support Palliat Care..

[10] Cosman ER (2005). A comment on the history of the pulsed radiofrequency technique for pain therapy.. Anesthesiology..

[11] Sluijter ME, Cosman E, Rittman III W, Kleef M (1998). The effects of pulsed radiofrequency fields applied to the dorsal root ganglion--A preliminary report.. Pain Clinic..

[12] Erdine S, Yucel A, Cimen A, Aydin S, Sav A, Bilir A (2005). Effects of pulsed versus conventional radiofrequency current on rabbit dorsal root ganglion morphology.. Eur J Pain..

[13] Cahana A, Vutskits L, Muller D (2003). Acute differential modulation of synaptic transmission and cell survival during exposure to pulsed and continuous radiofrequency energy.. J Pain..

[14] Bogduk N (2006). Pulsed radiofrequency.. Pain Med..

[15] van Kleef M, Barendse GA, Kessels A, Voets HM, Weber WE, de Lange S (1999). Randomized trial of radiofrequency lumbar facet denervation for chronic low back pain.. Spine (Phila Pa 1976)..

[16] Dreyfuss P, Halbrook B, Pauza K, Joshi A, McLarty J, Bogduk N (2000). Efficacy and validity of radiofrequency neurotomy for chronic lumbar zygapophysial joint pain.. Spine (Phila Pa 1976)..

[17] Pevsner Y, Shabat S, Catz A, Folman Y, Gepstein R (2003). The role of radiofrequency in the treatment of mechanical pain of spinal origin.. Eur Spine J..

[18] North RB, Han M, Zahurak M, Kidd DH (1994). Radiofrequency lumbar facet denervation: analysis of prognostic factors.. Pain..

[19] Tzaan WC, Tasker RR (2000). Percutaeous radiofrequency facet rhizotomy--experience with 118 procdedures and reappraisal of its value.. Can J Neurol Sci..

[20] Gallagher J, Di Vadi PLP, Wedley J, Hamann W, Ryan P, Chikanza I (1994). Radiofrequency facet joint denervation in the treatment of low back pain: a prospective controlled double-blind study to assess its efficacy.. Pain Clin..

[21] Ferrante FM, King LF, Roche EA, Kim PS, Aranda M, Delaney LR (2001). Radiofrequency sacroiliac joint denervation for sacroiliac syndrome.. Reg Anesth Pain Med..

[22] Cohen SP, Abdi S (2003). Lateral branch blocks as a treatment for sacroiliac joint pain: A pilot study.. Reg Anesth Pain Med..

[23] Sluijter ME, van Kleef M (1998). Characteristics and mode of action of radiofrequency lesions.. Curr Pain Headache Rep..

[24] Munglani R (1999). The longer term effect of pulsed radiofrequency for neuropathic pain.. Pain..

[25] Geurts JW, van Wijk RM, Stolker RJ, Groen GJ (2001). Efficacy of radiofrequency procedures for the treatment of spinal pain: a systematic review of randomized clinical trials.. Reg Anesth Pain Med..

[26] Mikeladze G, Espinal R, Finnegan R, Routon J, Martin D (2003). Pulsed radiofrequency application in treatment of chronic zygapophyseal joint pain.. Spine J..

[27] Lindner R, Sluijter ME, Schleinzer W (2006). Pulsed radiofrequency treatment of the lumbar medial branch for facet pain: a retrospective analysis.. Pain Med..

[28] Tekin I, Mirzai H, Ok G, Erbuyun K, Vatansever D (2007). A comparison of conventional and pulsed radiofrequency denervation in the treatment of chronic facet joint pain.. Clin J Pain..

[29] Geurts JW, van Wijk RM, Wynne HJ, Hammink E, Buskens E, Lousberg R (2003). Radiofrequency lesioning of dorsal root ganglia for chronic lumbosacral radicular pain: a randomised, double-blind, controlled trial.. Lancet..

[30] Shah RV, Racz GB (2004). Long-term relief of posttraumatic headache by sphenopalatine ganglion pulsed radiofrequency lesioning: a case report.. Arch Phys Med Rehabil..

[31] Marks RC, Houston T, Thulbourne T (1992). Facet joint injection and facet nerve block: a randomised comparison in 86 patients with chronic low back pain.. Pain..

[32] Van Zundert J, Lame I, De Louw A, Jansen J, Kessels F, Patijn J (2003). Percutaneous pulsed radiofrequency treatment of the cervical dorsal root ganglion in the treatment of chronic cervical pain syndromes: a clinical audit.. Neuromodulation: Technology at the Neural Interface..

[33] Ercelen O, Bulutcu E, Oktenoglu T, Sasani M, Bozkus H, Cetin Saryoglu A (2003). Radiofrequency lesioning using two different time modalities for the treatment of lumbar discogenic pain: a randomized trial.. Spine (Phila Pa 1976)..

[34] Shah RV, Ericksen JJ, Lacerte M (2003). Interventions in chronic pain management. 2. New frontiers: invasive nonsurgical interventions.. Arch Phys Med Rehabil..

[35] Sluijter ME (2000). The role of radiofrequency in failed back surgery patients.. Curr Rev Pain..

[36] Van Zundert J, Raj P, Erdine S, van Kleef M (2002). Application of radiofrequency treatment in practical pain management: state of the art.. Pain Pract..

[37] Abejón D, Reig E (2003). Is pulsed radiofrequency a neuromodulation technique?. Neuromodulation..

[38] Devulder J, Crombez E, Brusselmans G (2004). Comment on 'Pulsed radiofrequency treatment of the gasserian ganglion in patients with idiopathic trigeminal neuralgia'.. Pain..

[39] Orlandini G (2004). Pulsed percutaneous radiofrequency treatment of the Gasserian ganglion for therapy of trigeminal neuralgia: technical notes, validity of the method and selection of the patients.. Pain..

[40] Van Zundert J, Raj PP (2002). The technique of radiofrequency.. Pain Pract..

[41] Richebe P, Rathmell JP, Brennan TJ (2005). Immediate early genes after pulsed radiofrequency treatment: neurobiology in need of clinical trials.. Anesthesiology..

[42] Cahana A, Van Zundert J, Macrea L, van Kleef M, Sluijter M (2006). Pulsed radiofrequency: current clinical and biological literature available.. Pain Med..

[43] Gallagher RM (2006). Pulsed radiofrequency treatment: what is the evidence of its effectiveness and should it be used in clinical practice?. Pain Med..

[44] Cahana A (2005). Pulsed radiofrequency: a neurobiologic and clinical reality.. Anesthesiology..

[45] Jensen TS (2007). Pulsed radiofrequency: a novel treatment for chronic cervical radicular pain?. Pain..

